# A case report on the choice of venous access after ectopic peripherally inserted central catheter in an upper limb of a patient with a second recurrence of right-side breast cancer

**DOI:** 10.1097/MD.0000000000040104

**Published:** 2024-10-25

**Authors:** Tian Tian, Lin Tan, Hongyi Deng, Yi Lu

**Affiliations:** a Department of Vascular Access Nursing Clinic, People’s Hospital of Deyang City, Deyang, Sichuan, China; b Department of Nursing, People’s Hospital of Deyang City, Deyang, Sichuan, China; c Department of Radiology, People’s Hospital of Deyang City, Deyang, Sichuan, China; d Department of General Medicine, People’s Hospital of Deyang City, Deyang, Sichuan, China

**Keywords:** catheter malpositioned, innominate vein, intracavitary electrocardiography, lower extremity tunnel PICC, stenosis, TIVP

## Abstract

**Rationale:**

Intravenous catheter placement in the healthy upper extremity is preferred for chemotherapy in patients with breast cancer. Common venous accesses are peripherally inserted central catheters (PICCs) and totally implantable intravenous port catheters (TIVPs). In this case, a patient with breast cancer had a history of infusion port placement through the left internal jugular vein, with ipsilateral innominate vein stenosis after placement. The patient was re-treated with a PICC placed ectopically through the left upper limb into the intrathoracic vein. After multidisciplinary consultation, a transfemoral PICC combined with intracavitary electrocardiography (IC-ECG) was performed to establish venous access. This case can assist PICC catheterization nurses in developing optimal venous access strategies tailored to the specific situations of patients in similar situations. Through adequate evaluations and optimal selection of venous access, the success rate of disposable catheterization can be improved, and the risk of complications reduced.

**Patient concerns:**

A 53-year-old female with breast cancer had a history of infusion port, with ipsilateral innominate vein stenosis after placement. The patient was re-treated with a PICC placed ectopically through the left upper limb into the intrathoracic vein.

**Diagnoses:**

An axial computed tomography (CT) image before totally implantable venous access port (TIVP) placement and An axial CT 103 days after TIVP placement shows diffuse stenosis of the left innominate vein, associated with infusion port placement through the left internal jugular vein.

**Interventions:**

After multidisciplinary consultation, a transfemoral PICC combined with intracavitary electrocardiography (IC-ECG) was performed to establish venous access.

**Outcomes:**

The patient’s lower limb PICC was left in place for 201 days with no complications, completing the full treatment cycle.

**Lessons:**

This case presents a rare and insightful clinical scenario. For patients with a history of infusion port placement, particularly via the left internal jugular vein, careful analysis of the innominate vena cava and examination of chest wall vein exposure are essential to determining the optimal vascular access strategy.

## 1. Introduction

Totally implantable intravenous port catheters (TIVPs) and venous peripherally inserted central catheters (PICCs) are used to deliver chemotherapy in patients with breast cancer.^[1]^ TIVP is a safe and effective chemotherapy route for patients with cancer.^[2]^ The incidence of central venous stenosis is greater in patients with breast cancer after placement of TIVPs via the left internal jugular vein (IJV). The possibility of left innominate vein stenosis should be considered when a left TIVP needs to be placed in patients with right-sided breast cancer.^[3]^ Transfemoral PICC is a medium-to-long-term intravenous infusion with the catheter tip in the inferior vena cava (IVC) for patients without the option of a superior vena cava infusion.^[4]^ Intracavitary electrocardiography (IC-ECG) accurately locates the tip of the femoral vein catheter, minimizing the incidence of catheter-related complications and the time and cost of localization.^[5]^ We successfully established venous access for a patient with breast cancer and a history of left IJV access infusion port placement and ipsilateral innominate vein stenosis after placement. Re-treatment using a PICC ectopic to the internal thoracic vein via the left upper limb and a femoral vein tunneled PICC combined with IC-ECG positioning technology enabled the patient to complete the treatment cycle successfully.

## 2. Case description

A 53-year-old female presented with invasive ductal carcinoma of the right breast. After placement of a TIVP via the left IJV on June 8, 2016, and completion of 3 chemotherapy cycles with the FEC regimen (fluorouracil, epirubicin hydrochloride, and cyclophosphamide), the patient complained of swelling and discomfort in her left upper limb and left shoulder. Angiography suggested left IJV thrombosis (partial embolism). Four weeks after anticoagulation therapy, repeat angiography suggested that the thrombus was reduced significantly and local symptoms relieved. The TIVP was removed on September 19, 2016, after being left in place for 103 days. On August 31, 2018, the patient relapsed and was administered postoperative intensity-modulated radiotherapy to the tumor bed area of the right breast. She was discharged on October 19, 2018, after completing radiotherapy. The patient relapsed again on April 16, 2022, and received a PICC placement before chemotherapy. After evaluation and an ultrasound-guided PICC via the left upper limb VIP vein, the catheter was withdrawn to 22 cm after difficulty feeding the catheter to 32 cm; no blood return was seen when the catheter was pumped back in. After withdrawing from the catheter and supporting the guidewire for 5 cm, the catheter was returned to the original position and then moved to 42 cm. The blood was drawn back to normal, and no catheter echoes were seen in the bilateral IJVs on ultrasound. No P-wave changes were seen in the positioning of the IC-ECG, which was re-adjusted without any changes in the P-wave. After multidisciplinary team discussions (one radiologist, 1 interventionalist, 1 sonographer, 1 breast surgeon, and 3 specialist intravenous therapy nurses), ectopic catheter removal was provided based on the patient’s history of intravenous cannulation, the nature of the chemotherapeutic drugs, the duration of treatment, and the patient’s wishes. A right femoral vein–tunneled PICC combined with IC-ECG was performed to locate the catheter. The catheter placement proceeded without difficulty. The patient completed the treatment cycle successfully, and the catheter was removed on November 3, 2022. The catheter had been in place for 201 days without complications.

## 3. Discussion

### 3.1. Causes of malpositioned PICC

**Figure 1.**
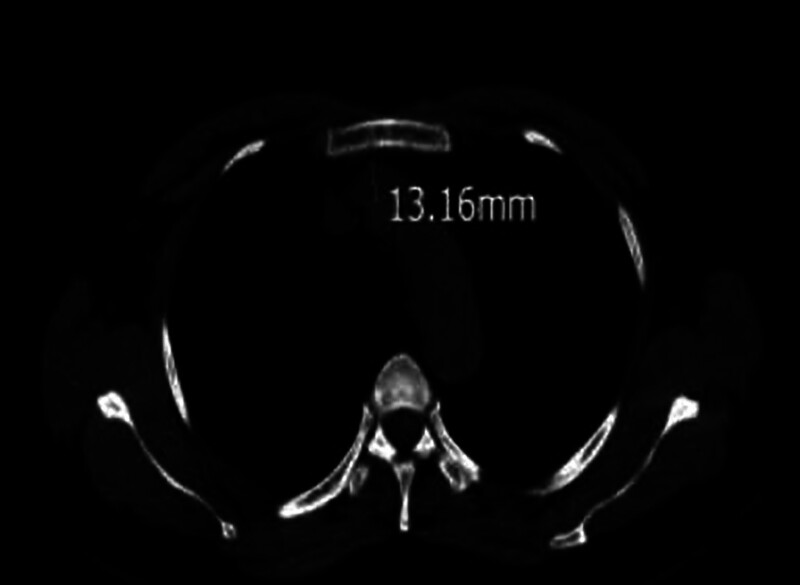
A 53-year-old woman with right-side breast cancer. An axial computed tomography (CT) image before totally implantable venous access port (TIVP) placement shows no evidence of left innominate vein stenosis.

**Figure 2.**
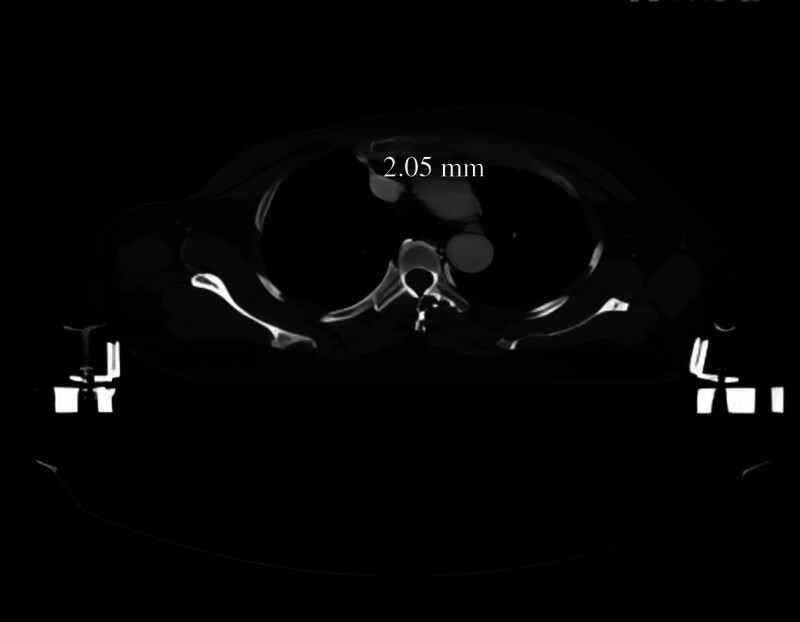
An axial CT 103 days after TIVP placement shows diffuse stenosis of the left innominate vein.

Hernández et al^[6]^ defined the criteria for venous stenosis as clear evidence of stenosis on venography (>30% narrowing of the vessel lumen diameter), with or without collateral circulation. Patients with a history of TIVPs placed through the left IJV have a greater incidence of central venous stenosis.^[3]^ Comparison of the patient’s 2 chest computed tomography (CT) scans revealed a 13.16 mm to 2.34 mm reduction in the innominate vein diameter before (Fig. 1) and after (Fig. 2) TIVP placement. The lumen diameter showed 82% stenosis; the imaging presentation was consistent with innominate vein stenosis, the most common cause of PICC malpositioning.

**Figure 3.**
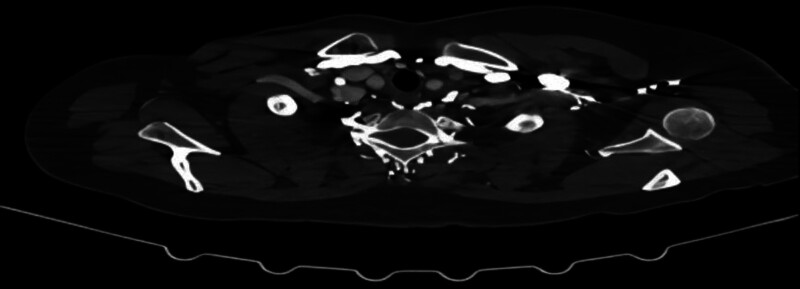
The development of multiple collateral vessels.

**Figure 4.**
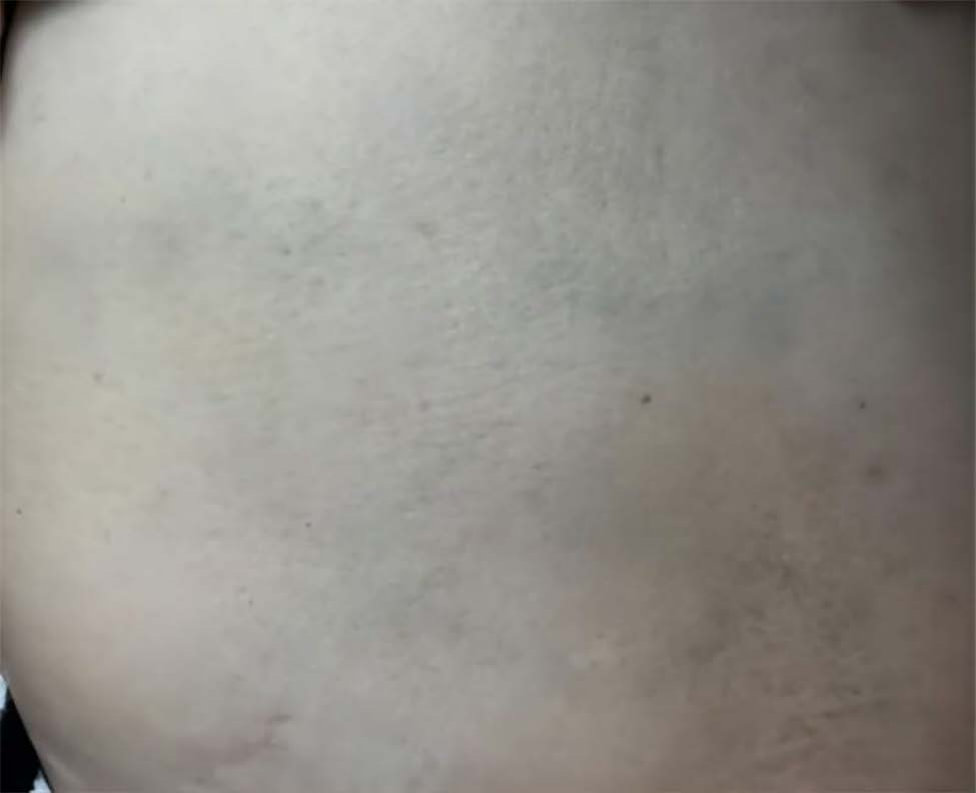
Static exposed vein in the left chest wall.

Partial or total narrowing of the vena cava lumen can obstruct blood flow and cause edema of the face, neck, and upper limbs, dyspnea, dizziness, headache, a varicose jugular vein, and exposure of the chest wall veins.^[7]^ In addition, as central vein stenosis gradually develops, it permits the development of collateral veins.^[3]^ Enhanced chest CT showed the contrast agent dispersed in all directions as it passed through the left innominate vein, creating several collateral veins (Fig. 3) and exposing the patient’s left chest wall vein (Fig. 4). The CT imaging images and the patient’s chest wall signs were consistent with obstructed blood flow following venous lumen narrowing. In cases of collateral vein formation, venous access is created for the PICC. These findings suggest that the PICC ectasia was caused by collateral vein formation.

### 3.2. Tunneled PICC placement

Tunneled PICC placement via the femoral vein combined with intracavitary electrocardiogram (IC-ECG) positioning is recommended for patients who cannot establish venous access in an upper limb. Annetta et al^[8]^ stated that ultrasound access to the femoral vein for central venous access is safe and feasible even in patients with low platelet counts or coagulation disorders. The tunneled PICC placement technique has advantages in minimizing PICC complications and improving patient comfort.^[9]^ Intraluminal cardiography accurately locates the tip of the femoral vein catheter, minimizing the incidence of catheter-related complications and the time and cost of localization.^[5]^ When the catheter tip is in the IVC, a low-blunted positive or negative P-wave is present; when it enters the right atrium, a bidirectional P-wave is present.^[10]^

**Figure 5.**
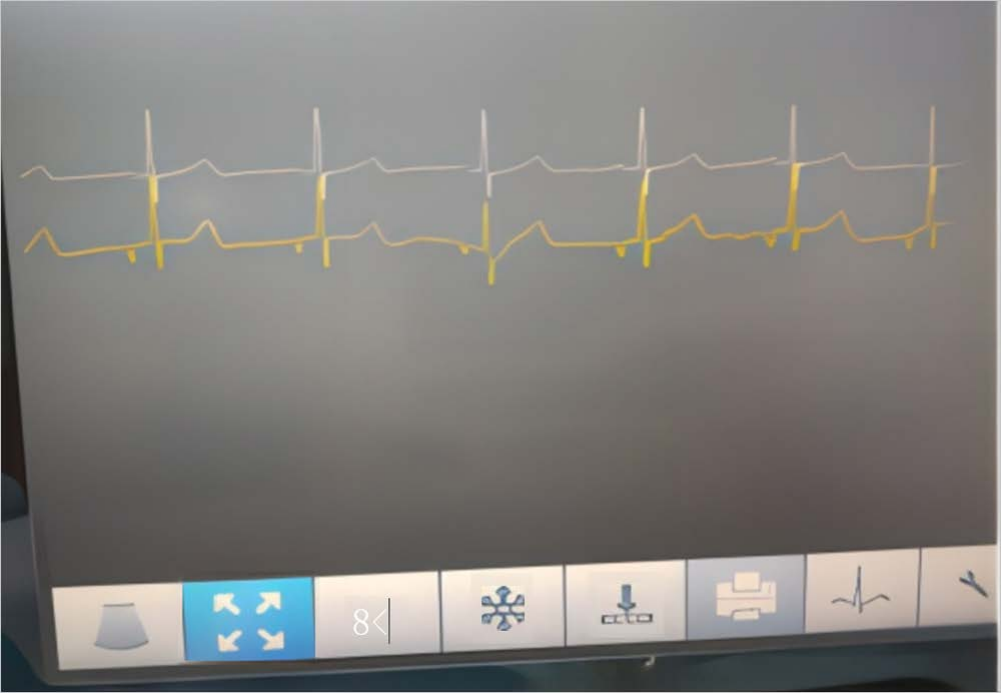
A negative P-wave is displayed when the catheter tip is in the IVC.

**Figure 6.**
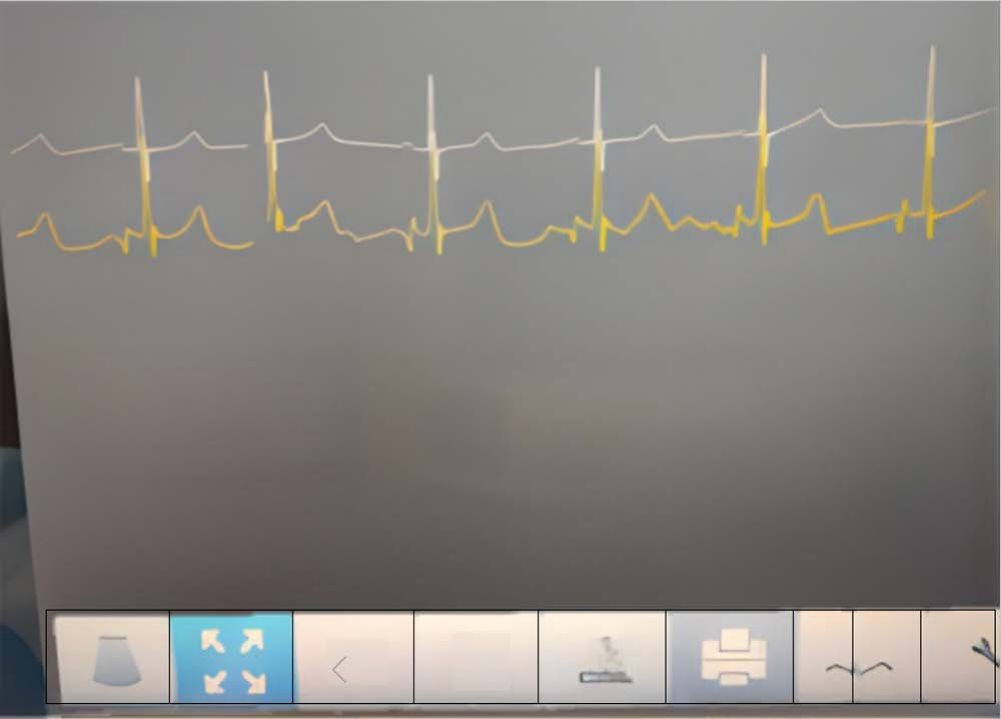
Bidirectional P-waves are displayed in the middle of the right atrium of the IVC when the catheter tip is in the middle of the IVC.

Wan et al^[4]^ suggested that the IVC PICC tip should be located below the renal vein at the lumbar 2 to 3 vertebral level to avoid catheter occlusion because of gravity or accidental insertion into the renal vein. This patient had an ectopic PICC placed in the left upper limb. After discussion with the multidisciplinary team, ectopic catheter removal was administered, followed by right femoral vein–tunneled PICC with IC-ECG positioning. This patient had an average surface ECG. After the catheter was fed for the predicted length, a negative P-wave appeared when the catheter tip was in the IVC (Fig. 5). Continuing to feed the catheter caused a bidirectional P-wave to appear when the catheter tip was in the middle of the right atrium of the IVC (Fig. 6). After the appearance of a bidirectional P-wave, the catheter was set back 2 cm, which was the final length of the indwelling endovascular catheter. An abdominal X-ray was performed to locate the catheter tip; the abdominal plain film suggested that the catheter tip had reached the lumbar 2 level. The patient’s lower limb PICC was left in place for 201 days with no complications, completing the full treatment cycle.

## 4. Conclusions

For patients with a history of port placement, especially via the left IJV, preplacement evaluation is critical. A CT scan of the chest not only provides a clear view of the anatomy of the innominate vien and superior vena cava but also accurately assesses these vessels for abnormalities such as stenosis and compression. The accuracy of the assessment is crucial to mitigate complications during placement, such as difficulty in catheter delivery and ectasia.

Additionally, assessing the patient’s chest wall vein exposure status is a key component of the pre-catheterization process. By evaluating the clarity, direction, and filling of the patient’s chest wall veins, the safety and effectiveness of the catheter placement can be better ensured.

Tunneled PICC placement via the femoral vein combined with IC-ECG positioning is recommended for patients unable to establish venous access in an upper limb.

While this case offers valuable insights, certain limitations remain due to its specificity. Therefore, readers should carefully consider these limitations and apply the findings with caution. In the future, we plan to further expand the scope of our study through multi-case comparative studies to enhance the generalizability and reliability of our findings. Additionally, we will focus on improving data collection and analysis methods to reduce the impact of subjectivity and bias.

## Acknowledgments

The authors are grateful to the patient for providing basic information.

## Author contributions

**Data curation:** Hongyi Deng, Yi Lu.

**Writing – original draft:** Tian Tian.

**Writing – review & editing:** Lin Tan.
